# Subspace Projection Approaches to Classification and Visualization of Neural Network-Level Encoding Patterns

**DOI:** 10.1371/journal.pone.0000404

**Published:** 2007-05-02

**Authors:** Remus Oşan, Liping Zhu, Shy Shoham, Joe Z. Tsien

**Affiliations:** 1 Center for Systems Neurobiology, Departments of Pharmacology and Biomedical Engineering, Boston University, Boston, Massachusetts, United States of America; 2 Shanghai Institute of Brain Functional Genomics, The Key Laboratories of Ministry of Education (MOE) and State Science and Technology Committee (SSTC), and Department of Statistical Mathematics, East China Normal University, Shanghai, China; 3 Department of Biomedical Engineering, Technion-Israel Institute of Technology, Haifa, Israel; Indiana University, United States of America

## Abstract

Recent advances in large-scale ensemble recordings allow monitoring of activity patterns of several hundreds of neurons in freely behaving animals. The emergence of such high-dimensional datasets poses challenges for the identification and analysis of dynamical network patterns. While several types of multivariate statistical methods have been used for integrating responses from multiple neurons, their effectiveness in pattern classification and predictive power has not been compared in a direct and systematic manner. Here we systematically employed a series of projection methods, such as Multiple Discriminant Analysis (MDA), Principal Components Analysis (PCA) and Artificial Neural Networks (ANN), and compared them with non-projection multivariate statistical methods such as Multivariate Gaussian Distributions (MGD). Our analyses of hippocampal data recorded during episodic memory events and cortical data simulated during face perception or arm movements illustrate how low-dimensional encoding subspaces can reveal the existence of network-level ensemble representations. We show how the use of regularization methods can prevent these statistical methods from over-fitting of training data sets when the trial numbers are much smaller than the number of recorded units. Moreover, we investigated the extent to which the computations implemented by the projection methods reflect the underlying hierarchical properties of the neural populations. Based on their ability to extract the essential features for pattern classification, we conclude that the typical performance ranking of these methods on under-sampled neural data of large dimension is MDA>PCA>ANN>MGD.

## Introduction

The emergent capabilities to simultaneously monitor the activities of over several hundreds of individual neurons in the brain [1]–[6] have vastly expanded the complexity of the resulting neural data sets. It becomes increasingly clear that traditional approaches that characterize first-order (e. g. peri-event rasters or peri-event histograms) or second-order (e. g. pair-wise cross-correlations and joint peri-event time histograms) statistics of time series of discrete spike trains (point process) are no longer adequate to deal with the complexity of the large data sets [7], [8].

In this paper we examine methods for categorical classification of discrete stimuli or episodic events based on the spike series responses they induce in a population of neurons. In addition, we focus on high dimensional, low sample sizes (trial repetitions) neural data, since many cognitive experiments may come with constraints in terms of allowing large trial repetitions. While several types of multivariate statistical methods have been used for integrating responses from multiple neurons, their effectiveness in pattern classification and predictive power have not been compared in a direct and systematic manner. In an attempt to provide empirical comparisons among those mathematical tools, we examine here the performance of a variety of multivariate statistical classification methods on standardized, representative data sets. The multivariate methods we study here include Multivariate Gaussian Distributions (MGD), which performs the classification task in the original high dimensional space, and Multiple Discriminant Analysis (MDA), Principal Components Analysis (PCA) and Artificial Neural Networks (ANN), which achieve classification by first projecting the original data sets into lower-dimensional subspaces [9].

Our empirical measurement of the performance of these methods are based on their classification scores of three data sets: 1) simultaneous recording of 250 neurons from the hippocampal CA1 region of mice subjected to episodic startling events such as acoustic startling sound, experimentally simulated earthquakes or free fall (elevator drop), and sudden air-blow to the back of the animal; 2) the simulated data sets of 250 neurons in the inferior temporal cortex of the monkey during presentation of face images; 3) the simulated data of 250 neurons in the monkey motor cortex during arm movements and rotation.

The experimental dataset from the mouse hippocampus can help us assess the performances of these methods in real world scenarios, whereas the simulated data sets enable us to numerically vary the neural correlations and noise levels in data modulated by categorical or continuous variables in order to to explore the effectiveness of these methods for pattern classification. We have also examined the potential complications when the trial/sample number is much smaller than the size of the analyzed neural population and how they can be addressed through the regularization methods. Another critical issue is how to select a classifier which would allow uniform comparison of the performances of these statistical techniques in order to determine which methods achieve more accurate classification on our data sets. Finally, we investigate the computations implemented by the projection methods by examining the detailed composition of the lower-dimensional subspaces.

## Results

**Figure 1:**
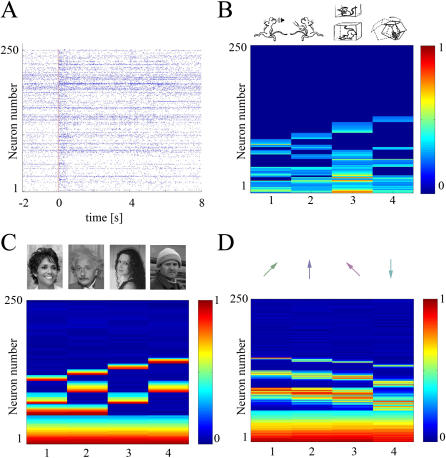
Neural population activities obtained from hippocampal recordings and simulated cortical responses to face presentation/arm movement. A) Neural population responses to a 30 cm drop are indicated by spike rasters of 250 simultaneously recorded CA1 hippocampal neurons starting from 2 s before and ending 8 seconds after the startle event, indicated by the vertical red line. B) Average frequency changes during the startle events indicate the existence of non-responsive neurons (48%) as well as neurons that respond to all startle (1–23), 3 types only (24–46), 2 types (47–85) or only to a single type of events (86–135) (the maximum change is normalized to be 1). The caricature images displayed on the top of the colormap correspond to each startle type: sound, air blow, drop and shake. C) Average responses of simulated neural responses to presentation of four human faces (famous face #1 Halle Berry, famous face #2 Einstein, non-famous face #3-female, non-famous face # 4–male). Out of a total of 250 neurons, we choose 50 to be responsive to all four faces, 20 to famous faces, 20 to the two female faces and 20 to the next two male faces. Finally, four populations of 10 neurons are assumed to respond to each individual face, with the rest of the 100 neurons being unresponsive. D) Simulated motor cortex population responses to arm movements at different angles *θ*∈{45°, 90°, 135°, 270°}; the stimulation angles are indicated by the green, blue, magenta and cyan arrows on top of the colormap. For ease of visualization neurons were sorted along the vertical axis by their tuning width, with 55 neurons responding to all faces, 40 and 30 units being more and less broadly tuned, respectively, and 25 units being very sharply tuned around their responsive angle, while the remaining 100 units are unresponsive.

We use the recorded hippocampal and simulated cortical data sets to illustrate the implementation of these multivariate statistical methods. For the hippocampal data, occurrence of a startle event produces significant changes in the population activity (Figure 1A). The average frequency changes during the startle events indicate that while a significant proportion of neural population (48%) does not respond to any startle stimuli, the remaining units changed their frequency after the occurrence of either all such types of events (9%), 3 types only (9%), 2 types only (15%) or only to a single type of event (20%) (Figure 1B). To facilitate direct comparison between different neurons, we used the transformation *R_i_* = |*f_i_*−*f*_0_|/(*g*_0_+*f*_0_), where f_i_ and f_0_ represent average frequency responses during startles of type **i** or rest states, and g_0_ is the average population activity during rest states. For the first simulated data set we assume that the neural responses are drawn from a neural population with a hierarchical structure, with neurons responding to presentation of any human face, to famous faces only, to male or female only, or to individual ones (Figure 1C). For the second simulated data set, we assume that the neural population is composed of units that are generally responsive to all arm movements, to movements at specific angles, or to movements that are sharply or broadly tuned around specific angles (figure 1D). While the neurons for the simulated data sets are modulated by either discrete or continuous variables, their responses are assumed to be drawn from Gaussian distributions.

### Implementation of subspace analysis classifiers via regularization

**Figure 2:**
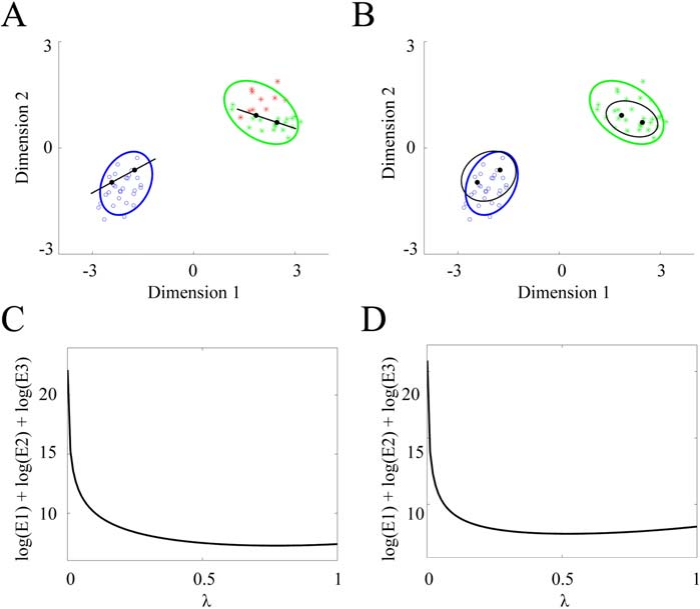
Regularization can prevent over-fitting of the training data sets. A) A two-dimensional example illustrate how a two-class classification between the two data sets (blue and green points drawn from two-dimensional gaussian distributions) is affected by selecting a small number of samples (two in this example). The probability distributions that best fit the selected points are too tight and introduces classification errors (red stars, for the green class). B) The probability distributions corresponding to the regularized covariance matrices yield better generalization and allow all data points to be classified classified correctly. The new 2σ boundaries are plotted in black for each class. Minimization of quantity F = log(E1)+log(E2)+log(E3), the log of error terms for self, opposite class and between-class separations, permits the selection of regularization parameters at the minimum for C) blue class at λ = 0.76 and for D) green class at λ = 0.52.

In practice, for data with large number of dimensions, the class covariance matrices are often ill-conditioned and non-invertible. As a result, the computed projection subspaces are potentially very sensitive to small variations of data. This is commonplace for sets represented in very high-dimensional spaces and a direct consequence of data under-sampling, as the dimension of the data points (number of recorded neurons) is much higher than the sample training data dimension (trial numbers). To illustrate the need for regularization, we employ a two-dimensional example which uses two distinct data sets drawn from Gaussian distributions (Figure 2A). An appropriate, if extreme, example of under-sampling is to choose two points from each class and to attempt to classify all remaining data points based on this choice. Obviously, the covariance matrix that best fits two data points corresponds to the line that unites them; this matrix has a zero determinant and is not invertible. The simplest example of such covariance matrix is a two by two array with all elements equal to 1, corresponding to perfectly correlated variables of equal standard deviations in both x and y dimension. Increasing the diagonal elements by a very small amount makes the covariance matrices usable in computations, although they are barely invertible. As a result, the generalized distance away from the center of the two-point point distributions is only slightly influenced by variations along the uniting lines and it is dominated by deviations along the orthogonal lines. Consequently, there is an increased chance of misclassification of test data points due to overemphasis of the best fit to the two-point sampled data sets (Figure 2A).

This singularity issue can be addressed directly by regularization of the covariance matrices of form: **Σ_i_' = (1−λ)Σ_i_+λ I** [10]–[12]. Here **λ** is a regularization parameter between 0 and 1, **Σ_i_** is the covariance matrix for the i^th^ class, and **I** is the identity matrix. As a result of regularization the determinant becomes non-zero and matrices **Σ_i_** can be now inverted. Previous research [12] used cross-validation results to search for the optimal regularization parameter. Here, we suggest an automatic way of selecting these parameters in the next paragraph, as this allows us to use the cross-validation results as an unbiased indicator of algorithm performances. Another solution used in the neural signal classification literature is to assume that all neural signals are uncorrelated, that is, to set the non-diagonal elements of the matrix Σ to zero, thus ensuring that its determinant is non zero [13], [14]. These methods may also require further regularization if the sampled neural population contains units with extremely low firing rates, which have mean and standard deviations close to zero; in this case the covariance matrix becomes also non-invertible. Another way of addressing this problem is through preprocessing, by eliminating such low-firing neurons or other units that have weak changes in firing rates between different stimuli paradigms [5].

The main consequence of the regularization methods mentioned in the previous paragraph is to increase the relative size of diagonal elements as compared to off-diagonal elements. We introduce the following choice for regularization: **Σ' = (1−λ)Σ+λ M I,** where **M** is the average of diagonal elements of the original covariance matrix **Σ**. The new ensuing probability distributions, obtained when using the ‘optimal’ regularization parameters, are shown in Figure 2B. These choices are made using the following procedure: for each regularized matrix the error terms for the points belonging to the self-class are first computed 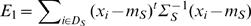, followed by the error terms for the points that belong to different classes 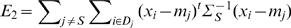 and for the between class centers 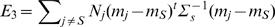. The regularization parameter **λ** is chosen in the region where the quantity **F = log(E_1_)+log(E_2_)+log(E_3_)** is minimized (see Figure 2C and 2D for an example). The rationale for this choice is to use information from different classes than own to choose regularizations parameters that are more likely to yield improved generalization. Obviously, the term E_1_ is minimized for **λ = **0 and monotonically increases with **λ,** as the covariance matrix **Σ** changes away from the best fit of the training data set. The terms E_2_ and E_3_ would likely decrease though as the covariance matrix **Σ** relaxes the correlation terms that renders it too rigid a fit for the self class into a more general uncorrelated matrix. This new matrix likely describes better both the data points that do not belong to the first class and the axis that unites the two classes; in practice these trends create a minimum for the quantity **F**.

### Class membership evaluation within the projecting subspaces

**Figure 3:**
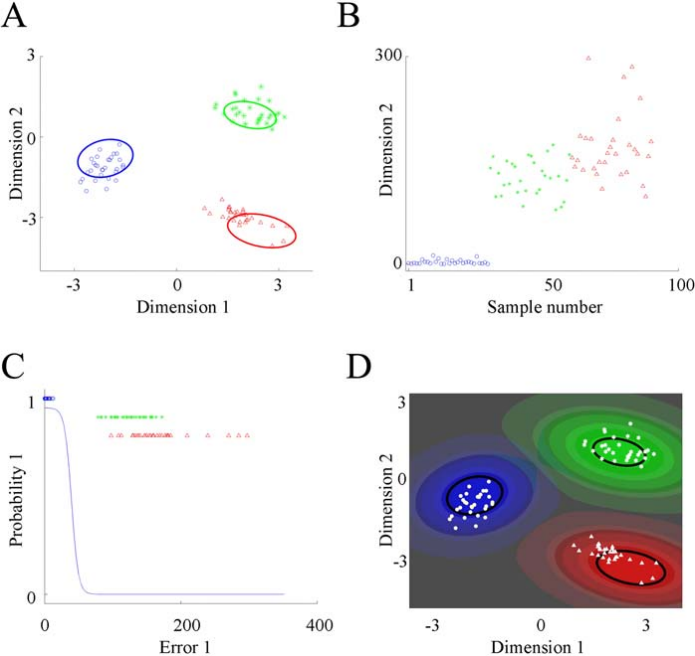
Class membership evaluation for a three-category example. A) Two-dimensional example illustrates the assignment to the closest cluster for a three-class membership (blue, green and red). We used the regularized covariance matrices to generate the 2σ boundaries for the under-sampled data points. B) The errors, or generalized distances away from the center of the blue cluster are plotted for all data points. Note that the blue points are characterized by low distances with a small standard deviation, in contrast to the green or blue points. The other error plots are similar in nature. C) Evaluation functions for determining the first class assignment. Data points with small and large errors are deemed to belong to the blue or other classes, respectively. D) Color plots indicating the distribution of probabilities throughout a region containing all data points. Each shade in the color gradation indicates an increase/decrease in probability above the chance levels (grey shade represents equal low probability for all three classes). The colors of the original data points and the original 2σ boundaries have been changed to increase their visibility.

Another essential technical issue pertaining to the application of these subspace projection methods is to determine how accurate the class membership evaluation is. A simple, non-parametric and efficient method to compute the class membership is to assign each data point to the closest cluster. This method, however, has the disadvantage of leaving the class probabilities undefined and of giving equal performance marks to points that are at different distances away from their corresponding assigned clusters. An example of these shortcomings is shown in Figure 3A, where class assignment is attempted based on the regularized covariance matrices of under-sampled data points from three two-dimensional classes. Distribution of the all data points errors (or generalized distances) for each particular class indicate that they are characterized by the small magnitudes and standard deviations for the self-class samples, and that the reverse is true for data points belonging to different classes (Figure 3B). This permits the use of the following probability evaluation functions for each class 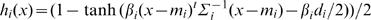, where d_i_ is proportional to the separation of self-errors and different-class errors and β_i_ = 4/d_i_. The choice for these probability functions is made such that the points belonging to the self class and all other classes receive probabilities close to 1 and 0, respectively, with a smooth transition as a function of generalized distance, as shown in Figure 3C. After computing all class probabilities for a particular point x, we normalize the sum of all probabilities. If the sum of probabilities is more than 1 (
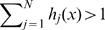
) we use multiplicative normalization 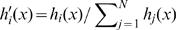 otherwise we employ additive normalization 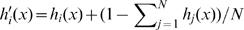 This particular choice of using both multiplicative and additive normalization has allowed us to compare algorithm performances uniformly. In particular, it is easy to envision particular data sets that have insufficient information to decide on the class membership; here all the data points have equal chance to belong to any class. In this situation, MGD, MDA, and possibly ANN, tend to use the noise from the large number of dimensions to produce tight fits for the training data points; consequently the test points are situated far away from the training cluster and receive low probability scores that do not add up to 1. Here the multiplicative normalization would select a class winner for MGD/MDA/ANN methods biasing them towards closest cluster selection, even when test point may be located quite far from all of clusters; in this situation additive normalization would be more appropriate as it assigns equal probability to all classes. In contrast, for the same data sets, the PCA method tends to produce larger size clusters, at times even overlapping, which yield similar probability scores for all test data points; here it is likely that total sum of probabilities is more than one, therefore of multiplicative normalization would yield class assignment similar to the other multivariate methods (MGD, MDA, ANN).

The use of the probability evaluation functions in conjunction with the normalization rules allow us to compute probabilities at any location in the low or high dimensional spaces (see Figure 3D for a two-dimensional example). Consequently, the evaluation of performances can be done by computing the probability that a test data point belongs to a class or another, as opposed to assigning it to the closest cluster. We note here that when class assignment is not the main goal of employing a particular multivariate statistical method, for example when used to monitor the dynamics in the low-dimensional projection subspace, different choices of normalization or even no normalization may be more appropriate.

### Evaluation of performance using both the experimental and simulated data

In order to facilitate a better understanding of the implementation of MDA, PCA, ANN, and MGD statistical methods and to evaluate their relative effectiveness in pattern classification, we use the experimental data sets from mouse hippocampus and the two simulated data sets from monkey cortex; all data sets contain 250 neurons and small number of repetitions of sampled stimuli. We first set out to compare the performance results on these three data sets by assessing the impact the neural variability has on the performances of the statistical multivariate methods.

For the electrophysiological data set, we have emulated an increase in the population noise level by identifying the units that are best classifiers and then systematically eliminating them from the neural population, thereby monotonically decreasing the overall signal to noise ratio. To estimate how good a classifier individual neurons are, we use the one-dimensional MGD probability distributions to compute the probability **h_ij_** that startle event **i** belongs to category **j.** Then an overall fitness score is computed: 
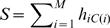
, where M is the number of instances in the training data set, and **C(i)** is the class membership of instance **i,** allowing us to obtain a sorted sequence of best discriminating neurons. To obtain a more accurate estimate of the overall performance of the statistical methods we created a collection of 8 test data points (4 data points for each stimuli as well as their corresponding 4 rest samples) to cross-validate the predictive power of models constructed using 48 training data points (24 rest samples and 6 data points for each one of the four classes). The mean performance is then obtained by averaging the results obtained for 100 different random training/test partitions.

**Figure 4:**
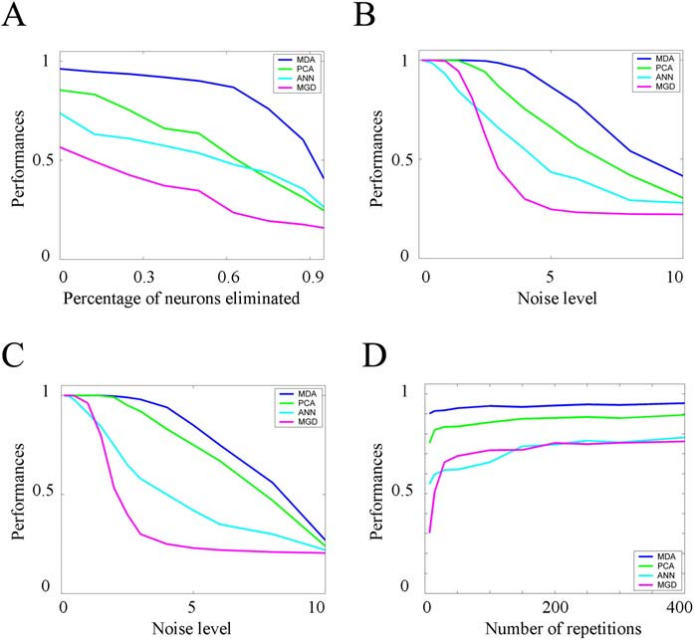
Performance evaluation for the multivariate statistical methods. A) The percentage of neurons eliminated (from best to worst single classifiers) is displayed on the horizontal axis, and the performances of methods are displayed on the vertical axis. The order of best performers is MDA>PCA>ANN>MGD. B) The accuracy of classification for face perception data set is plotted against the magnitude of the noise term. The relative ordering in test performances (MDA>PCA>ANN>MGD) is generally maintained among the different statistical methods with the exception of MGD which outperforms ANN at low noise levels. C) Results on the arm movement simulated data sets display similar trends to the face perception data set. D) Performances of all methods increase towards their asymptotical values as the number of sampled face perception training data points become larger. This increase of the training data set benefits most the lower performers, MGD and ANN, although their maximum performances do not reach the larger levels of PCA and MDA.

MDA performances for the test points from the electrophysiological data set indicate that eliminating the best neurons one by one to simulate an increase in the noise levels results in degradation of test data classification accuracy for all methods and that the sorted sequence of best performers is MDA>PCA>ANN>MGD (Figure 4A). When using the full number of neurons available, the performances of the methods were MDA = 0.96, PCA = 0.85, ANN = 0.74, MGD = 0.56. While performances for PCA, ANN and MGD decreased in relatively linear fashion, the MDA accuracy started decreasing in a significant fashion after approximately 50% of the best classifiers neurons are eliminated. This trend suggest the existence of a certain degree of redundancy in the information contained in the neural population, which allows MDA to maintain relatively stable performances in spite of the decrease in the signal to noise ration. The increased drop in performances as the size of sampled population is reduced towards small numbers also suggests the existence of a significant group of non-responsive neurons which does not contain information about the episodic events. We also note that the performances of all statistical methods degrade towards guess levels (20%) as the population is reduced to small numbers of unresponsive neurons.

We have further assessed the impact of the noise on the performances of the statistical multivariate methods by increasing the amount of neural noise on the simulated data sets, starting from small amount of variability which allows maximal performances for all statistical techniques and then increasing the noise levels gradually until all classification methods fail to perform better than random guess. For simplicity we also assume first that variability among neurons during exposure to a class of stimuli is uncorrelated. The data partitioning choice in the simulated data sets is made to emulate the choices made in the experimental data set. For each distinct level of the signal to noise ratio, the average performances for class prediction were obtained by computing the generalization performances of all statistical methods on 100 different training and test data sets.

For the simulated data set number one (face perception), we have found that all methods performed fairly well at the beginning (low noise levels), producing the performance sequence: MDA≈PCA>MGD>ANN (Figure 4B). As the noise levels increase, the performances of MGD are most affected and drop faster to minimal random guess levels; also the PCA methods are also more affected than MDA. At high levels of all performances are indistinguishable from the random guess levels. Thus, the analyses on this simulated data set have also led to the qualitatively similar performance-ranking sequence for the performance sequence: MDA>PCA>ANN>MGD. These trends are similar for simulated training data set number two (motor cortex data, Figure 4C).

We note that while in the experimental data set from the mouse hippocampus the performances for MDA method remain at relatively high levels until a significant fraction of cells is eliminated, the decrease in performances is more linear here as the level of noise increases. Also, when correlations between neurons are introduced for each stimuli class, the advantage the MDA has over the PCA method is increased (data now shown). Finally, we mention here that when dealing with data sets with large dimension, ANN, MGD, and MDA are perfectly capable of learning to classify the training data sets faithfully, regardless of the levels of noise; therefore the performances for the training data set are almost always close to 1. In contrast, the PCA performances are much more consistent between training and test data sets.

For a fixed level of noise, the performances of each method increase as the number of repetitions is augmented (Figure 4D, temporal cortex simulated data), as this allows obtaining better estimates for the center of multi-dimensional clusters and their covariance matrices. Addition of a few extra samples has the most pronounced effect when the size of original training data is small. In the asymptotical state, when the number of repetitions becomes large as compared to the number of variables, the performances of each method reflects how much they are affected by the variability in the neural data, producing the following ranking MDA>PCA>MGD≈ANN.

### Computation implemented by the projection methods

What are the main differences between these methods in terms of the computations that they are implementing? The ANN method constitutes a complex model, capable of computing a non-linear mapping between input and output, but the number of its underlying parameters is quite large, making it difficult to intuitively understand the computation they are implementing. MGD is not a projection method and all the information pertinent to classification is stored in large dimensional mean vectors and covariance matrices corresponding to each class. MGD computes the generalized distance from the center of the N-dimensional clusters corresponding to each class for all test point. Because each neuron contributes to this distance similarly, the noisy units can have a detrimental effect on the overall classification. As shown in the previous section, classification is facilitated by projecting into lower-dimension projection subspaces where the computations implemented by these subspace analysis methods essentially reduces to calculations of the weighting factors specifying the contribution of each original variables to the new dimensions. For the experimental data set and the two simulated data sets, the MDA and PCA computations are similar in nature, however, the cluster produced are usually tighter for MDA.

**Figure 5:**
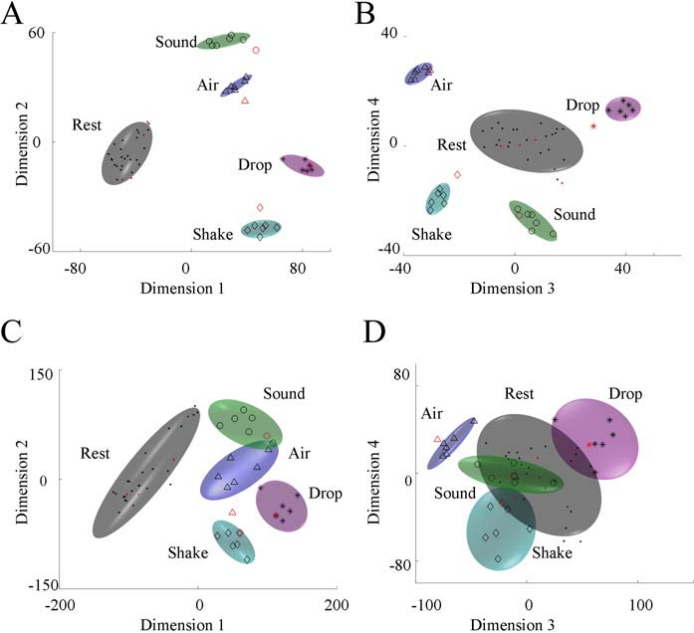
MDA and PCA projection subspaces for electrophysiological data set. A) First MDA dimension separates all startles from the rest data points (black dots), while the second dimension separates shake (diamonds) events from the metal sound events (circles). Training and test data points are represented in black and red color. Two dimensional gaussian distribution are fitted to the projected points for each class, revealing that they form segregated clusters (ellipsoids extend up to the 2*σ* boundaries). The colors black, green, blue, magenta and cyan denote rest, metal sound, air, drop and shake events, respectively. B) The third MDA dimension separates shake and airblow (triangles) classes from drop class (stars), and the fourth dimension separates metal sound and shake classes from drop and air blow classes. C) Inspection of the PCA discriminant dimensions indicate a similar structure of the encoding subspace with the first dimension separating the base from the rest of the startles and the second dimension separating the metal sound from shake. D) The third PCA dimension separates air blow from drop and the last dimension PCA separates shake from drop.

We previously showed [5] that a subpopulation of the recorded CA1 neurons responds to all types of startles, while other percentages of cells respond to either one type or to a combination of startle stimuli. The encoding MDA subspace seems to reflect the makeup of different types of cells, with the first dimension separating the startle classes from the base/rest state and the second dimension separating the shake from the acoustic startle (Figure 5A), while the third and fourth dimensions separate the drop event from the rest of the startling events and the air-blow event from the acoustic startle, respectively (Figure 5B). It is evident that the first two projections already separate the different classes into non-overlapping clusters and the last two dimensions further increase the global distance between these clusters. While the different classes separate analogously along different PCA projecting dimensions (Figure 5C and 5D), the clusters produced by MDA are smaller in size than the ones produced by PCA method. We note here that the performances for the PCA method can be improved by eliminating the neurons exhibiting weak startle responses over the base firing rates [5], as this improves the separation between base/rest class and the other classes.

**Figure 6:**
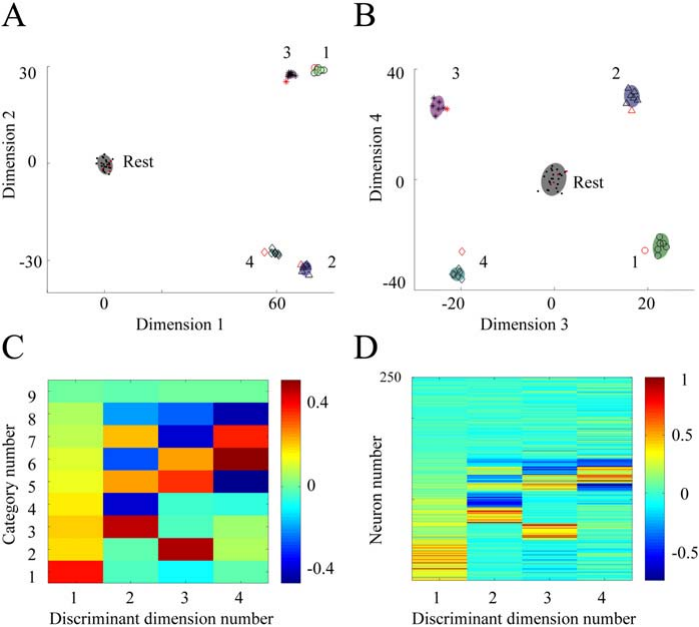
MDA projection subspace for the face perception cortical simulated data set. A) First MDA dimension separates the projection of neural responses to all perceptions from the projections of base firing rates (black). Second MDA dimension separates perception of female faces 1 (green, circles) and 3 (magenta, stars) from the male faces 2 (blue, triangles) and 4 (cyan, diamonds). B) Third MDA dimension separates famous faces 1 and 2 from faces 3 and 4. Fourth MDA dimension further increase the separation between classes 1 and 2 and between 3 and 4. C) Average weighting factors for MDA, with dimension number represented on the horizontal axis and with type of neurons represented on the vertical axis, starting from neurons responsive to all faces (line 1), to famous faces (line 2), to female faces 1 and 3 (line 3), male faces 2 and 4 (line 4), 1 only (line 5), 2 only (line 6), 3 only (line 7) and 4 only (line 8), and to non-response neurons (line 9) indicate that discriminating dimensions reflect the hierarchical structure used to generate the data. D) Individual weighting factors are also reflective of the different discriminating procedures implemented by these dimensions (general 1–50, famous 51–70, male 71–90, female 91–110, first face 111–121, second face 121–130, third face 131–140, fourth face 141–150, non-responsive neurons 151–250).

We also analyzed the simulated face perception in temporal cortex data sets to gain additional insight in what determines the makeup of the discriminate dimensions. Not surprisingly, the first MDA dimension separates all face perceptions from baseline activity, while the second dimension separates female faces 1&3 from male faces 2&4, with projection of the rest states lying in between (Figure 6A). The third dimension separate the perception of famous faces from perception of non-famous faces, while the last dimension uses information about each face perception to further increase their separation in the projecting subspace (Figure 6B). The PCA method exhibits similar trends.

Further inspection of the weight distributions for these dimensions reveals that they correlate with the magnitude of neural responses that satisfy the above-mentioned characteristics. The non-responsive neurons have no contribution to the first MDA dimension as their weighting factors have an average around zero (Figure 6C). The group of neurons that responds to all faces receives the largest weighting factors, followed by the less general neurons, responding to face category 1&2 (famous), 1&3 (female) or 2&4 (male), followed by the group of neurons that are specific to an individual face. For the second dimension, which separates the male and female faces, the non-responsive neurons as well as the most general neurons and “face famous neurons” have insignificant contributions. The neurons responsive to face perceptions 1, 3 and 1&3 have on average positive weighting factors, and the neurons responsive to perceptions 2, 4, and 2&4 have on average negative weighting factors. Moreover, the magnitude of the weighting factors for the neurons responding to highly specific individual faces is less than the neurons responding to two perceptions. On the third dimension the ‘famous face’ neurons become the larger contributors, followed by the individual famous-face neurons (1&2, positive contributions) and non-famous-face neurons (3&4, negative contributions). Finally, only the most specific neurons, responding only to single faces, further contribute for the dimension 4, increasing the separation between projected clusters. The individual weighting factors produced by the MDA subspace reinforce this view (Figure 6D).

**Figure 7:**
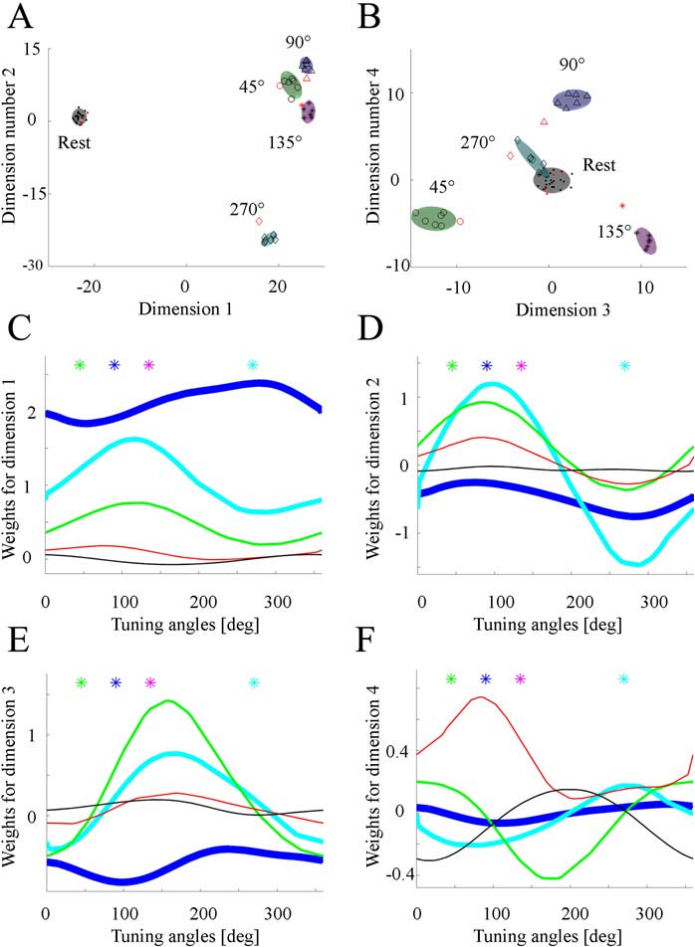
MDA projection subspace for the cortical arm movement simulated data set. A) First dimension yields separation between the black cluster (corresponding to the no-movement states) and the responses to arm movement at different angles (green for the 45°, blue for the 90°, magenta for the 135° and cyan for the 270° classes). Second dimension separates the forward movement clusters (45°, 90° and 135°) from backward movement cluster (270°). B) Third dimension separates the left and right directions (green 45° from magenta 135°), while separation between forward (blue 90°) and lateral-forward (green 45° from magenta 135°) is attempted in the last dimension. The generalization performances for these dimensions correlate with the magnitude of the corresponding eigenvalues sequence generated by MDA: 0.74, 0.16, 0.06 and 0.03 (sum of all eigenvalues is normalized to 1). C) Smoothed weight distributions on the first dimension indicate that neurons responsive to all movements have the largest contribution to this dimension (thick blue line) followed by broadly tuned neurons (thick cyan line) and sharply tuned neuron (green line). Neurons sharply tuned to a specific angle (thin red line) and non-responsive (thin black line) units have very small contributions. Stimulating angles (green 45°, blue 90°, magenta 135° and cyan 270°) are listed as colored stars symbols on the top of the plot. D) In the second dimension the broadly tuned neurons have the largest contributions, followed by sharply tuned and angle-specific neurons, with the rest of the units having negligible contributions. E) Third dimension, which separates left from right, relies more on the sharply tuned units and less on the broadly tuned units, with minimal contributions from the rest of the neurons. F) Inspection of the fourth dimension curves indicate that the very sharply tuned neurons are used in the attempt to separate forward from oblique movement.

Similarly, classification of neural activity in the motor cortex during resting states and arm movements at four different angles can be solved in a four-dimensional discriminating subspace, where all classes of population responses are projected to segregated regions. In this subspace, the first dimension yields maximum separation between no-movement class and the other classes (Figure 7A), while the second dimension complements the first dimension by providing separation between classes corresponding to *θ*∈{45°, 90°, 135°} and *θ* = 270° (forward and backward movements). The third dimensions of the projection subspace separates left and right directions, for *θ*∈{45°, 135°} (Figure 7B), while the fourth attempt to separate *θ* = 90° from *θ*∈{45°, 135°}. Out of the 4 projections generated by MDA, the first two dimensions account for most of the variations in the data set. Interestingly, inspection of the linear weights for the first dimension reveals that the units that are generally responsive to movements have a larger impact on deciding on the existence of movement than the other angle-tuned units (Figure 7C). In addition, the second dimension implements a computation of the vertical axis components which rely more on the broadly-tuned units and less on the sharply-tuned ones (Figure 7D). This sequence is reversed on the third dimension, which implements an oblique left versus oblique right decision, and the more sharply-tuned neurons have more impact than their broadly-tuned counterparts (Figure 7E). Finally, separation between forward and oblique movements is attempted in the fourth dimension (Figure 7F), mostly by relying on the very sharply tuned angle-specific neurons, but the generalization power of this computation suffers if the number of such sampled units is small. The structure of weighting factors for the discriminant dimensions suggests that although the arm movements have been sampled at only four discrete angles, the computation implemented by the projection methods takes advantage of the continuous modulation of neurons by pooling units with similar tuning properties to achieve discrimination between different types of movements.

## Discussion

The emergence of high-dimensional datasets poses challenges for the analysis and identifications of dynamical network patterns, because many traditional statistical tools may lose their efficiency dramatically as the numbers of dimensions increases. While several types of multivariate statistical methods have been used for integrating responses from multiple neurons, their effectiveness in pattern classification and predictive power has not been compared in a direct and systematic manner.

To address this issue, we have uniformly compared the performances of different multivariate statistical methods on recorded and simulated data sets, and assessed to what extent reducing the complexity of the initial encoding space improves the classification task. We have further investigated their robustness by evaluating their performances as the amount of noise in the data is increased and we conclude that for our experimental and simulated under-sampled high-dimensional neural data the order of best performers is MDA>PCA>ANN>MGD. While we believe that the ranking should have general implication because the analyses of three large datasets are quite representative of three major areas of neuroscience research (namely, learning and memory in the medial temporal lobe, motor controls and planning in the premotor and motor cortex, and visual information processing in the visual cortex), we acknowledge that at this stage we can not completely rule out the possibility that the ranking sequence might not be the exactly same for all future large datasets. We also note here that while PCA methods have similar performances between performances on training and test data, MDA and MGD require regularization to prevent over-fitting of training data sets. Prevention of over-fitting can be done by stopping the training of ANN early, but it is not clear how this can be done optimally. In addition, the implemented mapping between the input and output layer may not reflect the intrinsic structure of the data; these features may prevent the ANN methods from achieving better performances. More insight into the ranking of these methods could be obtained by studying the theoretical bounds for their performances, for example by computing their performances using the correct centers of the clusters and covariance matrices used to generate the training data.

In addition, we have looked into the computations that are implemented by the projection methods, primarily focusing on MDA and PCA. We note here that these methods are ideal to address categorical classification of data set. If the sampled data is continuous in nature (e. g. Local field potential, Electroencephalography, Functional magnetic resonance imaging) other methods, such as Independent Component Analysis (ICA), may be better suited for separating the continuous signals into independent components [15], [16].

**Figure 8:**
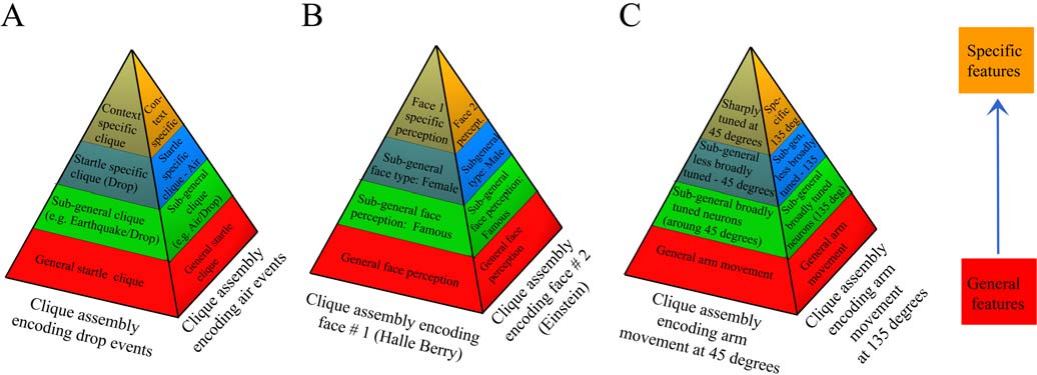
Hierarchical and categorical structure in the populations of neurons encoding: A) Episodic events, B) Face perception, and C) Arm movement. The neurons at the bottom of the hierarchical pyramid represent a common encoding block with broad tuning properties, responding to the most general and abstract features. The next layers of encoding neurons respond to sub-general, yet still multi-feature, properties of the episodic/perception/movement events. The neurons at the top encode the most specific features, allowing for the highly specific discrimination of a particular event, face, or movement direction.

MDA is a supervised dimensionality-reduction method that attempts to obtain maximum separation between neural population responses corresponding to different types of known stimuli by identifying and integrating the classification-significant neural features [5]. The PCA technique is another widely used projection method that has been used to explore regularities in the data set [5], [17]–[19]. Although the PCA projection method does not take into account the class membership, and is therefore constitutes an unsupervised method, the low-dimensional encoding subspace generated by the first few eigenvectors is typically also effective at separating the different classes. Due to its unsupervised nature, the PCA method tends to be less affected by overfitting than the MDA method, yielding similar performances for the training and test data sets. These two eigenvalue/eigenvector techniques are very useful for extracting the essential features down to an encoding subspace of lower dimensionality [5], [18], [20], allowing for easier visualization and monitoring of the recorded dynamics. Being linear in nature, they may be the first methods employed in attempting to find low-dimensional classifying subspaces embedded in the higher-dimensional data spaces. In addition, our analyses indicate that the computations implemented by these methods reflect the underlying hierarchical and categorical structure within the neural populations (Figure 8).

The selection of the input data can have a profound impact on the performances of the statistical methods. For example, while the duration of hippocampal neural responses to startling episodes ranges from a few hundred milliseconds to tens of seconds, the majority of such responses are within one second. As such, a selection of a too-narrow time-window as small as 10 ms would not capture critical details of these responses, as it would cut off a large part of the relevant spike responses. On the other hand, selection of a time window as large as 10 seconds would be too large, as the majority of the neurons would have returned to the base firing rates after a few seconds. Previous research in primary sensory and motor regions suggests that multivariate methods perform better when the bin sizes used to create the input data sets have a width above 100 ms [21]. For our data sets we choose to partition a one-second time interval in two bins, to include information about the initial activation and the subsequent sustained activity, while choosing time bins large enough to be robust to time variation that may be caused by delays in responses after the startle stimuli. A more detailed partition, of three or more bins in the 1 second interval could improve the classification performances, however, as the dimension of the original subspace grows, the computation becomes increasingly under-determined. For our actual experimental data, we found that using a time window of at least 750 ms and time bins with widths between 250 and 500 ms yields similar optimal performances for the test data sets prediction.

Under-sampling of relevant stimuli due to experimental constraints, such as small number of stimuli repetition to avoid behavioral habituation, poses another major problem for the multivariate statistical methods, because the covariance matrices describing the each class distribution become singular. As shown in the applied statistics studies, regularization can address this problem directly [10]–[12], although it is noteworthy to point out that there is little research about how to find out the optimal parameters for regularization. Too little regularization is only marginally helpful by making the covariance matrices barely invertible and still too rigid a fit for the original distributions, while too much regularization can erase the correlation coefficients from the original distribution, losing potentially useful information. One automatic regularization procedure found in literature to this problem is to the search for solution of equation of form **Σ x = b** (which involves a formal inversion of matrix **Σ**), which can be achieved by slightly incrementing all diagonal elements in Σ by a constant factor (Tikhonov regularization, [22]). The optimization problem can now be defined as finding the vector **x** that minimizes the quantity **||Σ x−b||^2^+λ^2^ ||x||^2^**, where **b** is a column vector, **||x||** indicates the norm of **x,** and **λ** is a positively defined regularization parameter. An appropriate choice for the **λ** values should be a good compromise between too little (**λ = 0**, overfitting) or too much filtering (λ large), and earlier research [23], [24] suggest that appropriate values lie at the corner of the so-called L-curve plot that displays the **log(||Σ x−b||)** vs. **log(||x||).** While this method provides automatic selection for the regularization parameters, it is not clear what would be an appropriate choice for the column vector **b** and to what extent the optimization obtained for this inverse problem yields optimal results for the classification problem.

We describe a new way of performing the regularization step that allows us to select a parameter in such a way that it improves the generalization, by looking for minimization of class errors not only for the self-class, but also for other classes and in-between classes. This method performs fairly well for both the experimental and simulated data sets, yielding increased generalization performances for MDA and MGD methods described here. Furthermore, these results are robust to choices of the regularization parameters, as the functions that determine them change smoothly around their minimum region where these parameters are selected.

The existence of embedded signal and noise subspaces also plays an important role in determining the successful application of the multivariate statistical methods. In general, the use of projection methods is closely related to the problem of classification, where the projection subspace dimensions are used instead of the initial variables to discriminate between two or more naturally occurring groups or events. In the context of the neural population recordings, the differentiation between these groups or events is usually based on quantitative information on the neural responses to external stimuli as well as the internal state of the brain. Consequently, it is often the case that the ensemble response space is composed of embedded signal and noise subspaces. The existence of a lower-dimension signal subspace is supported by the fact that many of the recorded neurons are encoding a small number of features about the external inputs, acting as linear or non-linear filters. In addition, in order to simplify alternative interpretations of the results, the experimental designs are normally trying to change a few environmental parameters at a time. The methods discussed in this paper focus on discrete methods, however, even responses that are modulated by continuous variables can still embedded in a lower dimensional subspace [25], [26]. The noise subspace accounts for variability in neural responses in response to stimuli or it may reflect changes in the internal states that are unrelated to changes in experimental conditions. In the signal subspaces created by the MDA or PCA methods the first dimensions usually contain more classifying information than the remaining ones, depending on the decreasing sequence of eigenvalues, providing a natural way of separating the signal components from the noise ones. In contrast, for the MGD method all dimensions contribute equally to classification and thus this method does not provide such separation of signal and noise subspaces.

The problem of over-fitting can potentially be a real concern for the supervised techniques (MGD, MDA, ANN) and evaluations of their performances are more accurate when using cross-validation procedures. As a consequence, non-supervised methods such as PCA are more appropriate for an unbiased search of regularities in the data set, have greater power of generalization and potentially can reveal underlying patterns in the neural data. However, when the classes to be separated are intrinsically different in nature, supervised methods, such as MDA, outperform the unsupervised ones, by automatically searching for the features that best differentiate different classes. Based on our analyses, it seems to be preferable to use MDA over PCA for experiments where distinct types of stimuli are delivered in a controlled fashion; on the other hand, PCA has better potential when used as an exploratory tool for searching the underlying similarities in the neural signals as its performances are more consistent between training and test data than the ones generated by the MDA methods. Thus, we recommend the use of both supervised and unsupervised methods to confirm the pattern classifications [5].

The dimensionality-reduction methods can also facilitate visualization of the ensemble neural activity patterns by projecting the data in a low-dimension encoding subspace. In addition, these dimensionality-reduction methods can be employed to investigate the dynamics of the neural patterns outside the window of time used to define the training/test patterns. By making use of the linear weighting factors corresponding to the projection dimensions, one can project the instantaneous neural frequencies within a moving sliding window (e.g. 1 second width) to the low-encoding projection subspace, enabling the direct visualization of the temporal evolution of dynamic activity patterns throughout the whole experiment. The application of sliding window technique to MDA has led to the direct visualization of encoding and reactivations of ensemble traces during formation of episodic memory [5], [20], (see also [27], [28]).

In conclusion, we have systematically compared the performance of several multivariate statistical methods on large neural data sets and we have found the following ranking MDA>PCA>ANN>MGD. Our empirical analyses of both experimental and simulated data suggest that, after addressing the under-sampling problem, the linear eigenvalue/eigenvector methods (MDA and PCA) are particularly effective for extracting essential features from complex data sets of large numbers of simultaneously recorded neurons and for visualization of underlying activity patterns and dynamics at the neural network level.

## Materials and Methods

Based on several multivariate statistical tools reported in the literature for visualization and classification of high dimensional data [5], [9], [17]–[19], [29], we use the most well-known methods such as MGD, MDA, PCA, and ANN. These multivariate statistical methods are described below.

### Multivariate Gaussian Distributions (MGD)

The class membership can be estimated directly in the original high-dimensional space of neural firing rates by computing the Multivariate Gaussian Distributions (MGD) 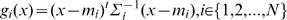, also known as Normal Density Discriminant Functions [9]. Here **N** is the number of dimensions (neurons), **x** is the population response treated as an **N**-dimensional point **x = (x_1_, x_2_, …, x_N_)**, **m_i_** is the mean response to stimuli for class ***i,*** **x^t^** is the transpose of vector **x**, and 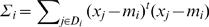 is the covariance matrix of the i^th^ class, which contains the set **D_i_** of stimuli repetitions. Each MGD function calculates the generalized distance from the center of a cluster, which in turn can be used to compute an approximate class membership evaluation by assigning each data point to their closest cluster or by using a classifier which computes the probabilities for each class. The performance of this method is then evaluated on test data points that are not included in the training data set.

### Multiple Discriminant Analysis

Multiple Discriminant Analysis (MDA) is a supervised statistical method that can be used to separate neural responses corresponding to different stimuli paradigms into distinct classes [5], [30], [31], (also known as Canonical Discriminant Analysis [32], or Quadratic Discriminant Analysis [10]–[12]). The mean responses during rest and activated states are computed for the training data and used to calculate the between-class scatter matrix 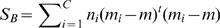. Here **C** is the number of classes, **n_i_** is the number of elements in each class, **m_i_** is the mean frequencies vector for each class and **m** is the global mean frequencies vector. The discriminant projection vectors are obtained as the first resulting eigenvectors in an eigenvalue decomposition of the matrix product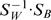 where the within-class scatter matrix **S_W_** is defined as: 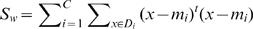, with **D_i_** representing the set of population responses corresponding to the **i** ^th ^class type. For theoretical reasons, this eigenvalue decomposition produces at most **C-1** non-zero eigenvalues, thus providing an upper bound for the dimensionality of the resulting projection subspace*.* In general, the first dimension exhibits the most separation between classes, but the subsequent dimensions may further increase the global separation, thus improving the overall discrimination, although by a lesser and lesser extent. Assessing class memberships in the low-dimensional space can be done by computing the MGD functions in the projected low-dimensional subspace and using them to evaluate the membership probabilities of each test point.

### Principal components analysis (PCA)

The PCA technique is an unsupervised method that attempts to capture most of the variance observed in the original data set by computing the eigenvectors of the total scatter matrix: 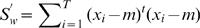, with 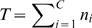 being the number of all the trials across the entire experiment [17]–[19]. We note here that the PCA scatter matrix is different from the one created by MDA, since in this case the training data is not partitioned into different classes. Evaluation of the performances is done in a similar fashion to the MDA method, by evaluating the class membership using the MGD functions in the low-dimensional subspace. In the data sets analyzed in this paper, the magnitude of the eigenvalues corresponding to the set of eigenvectors decreases dramatically after the first few dimensions and the number of relevant dimensions for both MDA and PCA subspaces is comparable most of the time. Therefore, in order to be able to compare their performances uniformly, we evaluated the performances in a PCA encoding subspace of dimension equal to the MDA subspace.

### Artificial Neural Networks (ANN)

The Artificial Neural Networks (ANN) employed in this paper are feed-forward Multi-Layer Perceptrons with back-propagation training (MLP, [9], [33]). They are capable of performing complex non-linear mapping between the training data and their class membership, and they have been successfully used to analyze neural datasets [34]–[36]. The type of ANN used here has one input layer, two hidden layers and an output layer, with transformation function between layers of smaller and smaller size being logsig, logsig and linear, respectively (***logsig(x) = 1/(1+exp(−x))***). This type of computation can be interpreted as successive transformations of the multidimensional input ***X*** into outputs of reduced dimensionality. The neural network is trained to compute the class membership by adjusting the values of the linear connection weights between its layers. This supervised learning method back-propagates the error term 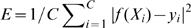
from the output layer towards the input layer in order to perform a gradient descent search for the optimal set of parameters that map the input to the corresponding class membership. Here |*X*| is the norm of input vector ***X***, **f(X)** is the computation implemented by the ANN and **C** is the dimensionality of the output layer. The gradient search is not guaranteed to reach global minimum and it can also lead to over-training, therefore requiring evaluating performances on the test data set.

For the electrophysiological data set we used a feed-forward neural network model from the Matlab Neural Network toolbox with 500 nodes as input, 200 nodes in the first hidden layer, 50 in the second one and 5 output units. The number of units in the input and hidden layers is smaller by a factor of approximately two (250 for the input layer and 100 and 25 for the hidden layers) for the simulated data sets.

### Experimental data set

We obtained experimental data by simultaneous recording of as many as 250 CA1 neurons in response to four types of startling memory events encountered by mice, described in Lin et al. [5], [20]: A short and loud acoustic startle (intensity 85 Db, duration 200 ms), 2) A sudden air-blow to the animal's back (termed Air-Blow, 200 ms, 10 p.s.i); 3) A sudden drop of the animal inside a small elevator (termed Elevator-Drop, vertical freefall height from 40 cm); and 4) A sudden shake-like cage oscillation (termed Shake, 200 ms; 300 rpm). The startle stimuli were triggered with the use of a computer at randomized intervals on the order of a few minutes. The stimuli were repeated 7 times per session to obtain a better sampling of the neural responses. Each session typically lasted around 30 minutes and the duration of the entire experiment was between 4 to 6 hours. An example of neural responses to the elevator-drop stimuli is shown in Figure 1A.

In order to ensure that the input data captures the dynamics of neural responses during the startle responses, we focused on a time window of a few seconds centered on the occurrence of the startle event. We used pre and post-event time intervals to obtain estimates for the base and startle responses firing rates. As a first-order approximation for the temporal variations of neural responses to startle stimuli, namely rising time, maximum amplitude and decay time, we split the sampling 1 second time intervals into 2 bins of 500 ms width, and we used the spike counts in each bin as an estimate for the neural population activity. The population neural response to a startle stimuli ***S*** can be formally described then by the population vector: *X_si_* = (*X_i_*_1_, *X_i_*_2_,…, *X_iN_*), where ***X_ij_*** is the frequency response of neuron ***j*** for the ***i*** ^th^ repetition of stimuli ***S*** and ***N*** is the number of binned frequency responses. For the data set presented in Figure 1A, when the period of interest is 1 second and the number of bins is two, it follows that the dimension of vector X_Si_ is twice the number of recorded neurons, N = 2•250 = 500. As a first approximation, the responses to stimuli *S* can be then characterized by the mean population responses 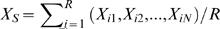, where R is the number of repetition (R = 7), and by their standard deviations. Analysis of the spike-raster plots and peri-event histograms of the neural responses to the four types of startle stimuli indicates that a subset of the recorded CA1 units is sensitive to all four types of startling events, whereas other cells appeared to respond to either air blow, drop, shake or sound alone, or to a combination of two or three different types of startles. This categorical and hierarchical organization seems to be a general property that exists across individual animals [5], [20]. The existence of these subpopulations suggests that at the CA1 level the startling events are represented by activity patterns of unique assemblies of neural cliques organized in a hierarchical and categorical manner (Figure 1B).

### Simulated data set 1–face perception in temporal cortex

Based on the studies of single-unit recordings of face-selective cells in the monkey inferior temporal cortex [37]–[40], we simulated the responses of 250 simultaneously recorded neurons in the inferior temporal cortex that respond to visual presentations of human faces. We assume that we can monitor the base firing rates of the neural population *X_basej_* = *a_j_*+*η_j_*, *j*∈{1, 2,…, *N*} (N = 250), as well as their responses to the presentation of four types of human faces *X_ij_* = *b_ij_*+*η_j_*, *i*∈{1, 2, 3, 4}. Here ***a_j_*** represent the base firing rates, ***b_ij_*** are the magnitude of maximum increase/decrease of frequency responses of neuron ***i*** during the ***j^th^*** face presentation and *η_j_* is the noise term. As it appears that neural responses might be also described by a hierarchical representation [38], [40], we choose an example supporting this view, with neural subpopulations responding to either single perceptions or multiple selective combinations of face perceptions (See illustration in Figure 1C). The general categories illustrated in this example are general responses to any face presentations, or responses to male, female or famous face presentations.

### Simulated data set 2–neural responses to arm movements in the motor cortex

Our second simulated data set consists of motor cortex population responses associated with arm movement in different directions [25], [41]–[43]. We assume that we can simultaneously monitor the activities of 250 individual neurons during arm movements at four different angles. The motor cortex neural responses to arm movement along different directions are assumed to be described by *X_ij_* = *a_j_*+*b_j_* *G*(*θ_i_*−*θ_j_*, *σ_j_*)+*η_j_*, where ***a_j_*** represent the base firing rates, ***b_i_*** are the magnitude of maximum increase/decrease, *θ_j_* is the preferred angle of neuron ***j***, and *η_j_* is the noise, while *θ_i_* is the angle for ***i*** ^th^ direction. Here we assume that the tuning curves *G*(*θ_i_*−*θ_j_*, *σ_j_*) are Gaussian functions with widths σ_j_. We construct data sets with a hierarchical structure by assuming that the neural population is composed of units from one the following five classes: unresponsive, generally responsive, broadly tuned, sharply tuned and angle-specific units. An example of such data set is illustrated in Figure 1D.
